# Prevalence of Dental Anomalies and Its Role in Sex Estimation among Children of Jazan Region, Saudi Arabia

**DOI:** 10.3390/children10040759

**Published:** 2023-04-21

**Authors:** Apathsakayan Renugalakshmi, Thilla Sekar Vinothkumar, Ahmed M. Bokhari, Samaher Almahdi, Abdulrahman Almalki, Sudheer Babu Balla, Santosh Kumar Tadakamadla, Zaki Hakami

**Affiliations:** 1 Division of Pedodontics, Department of Preventive Dental Sciences, College of Dentistry, Jazan University, Jazan 45142, Saudi Arabia; 2 Department of Pedodontics, Saveetha Dental College and Hospitals, Saveetha Institute of Medical and Technical Sciences, Chennai 600077, India; 3 Department of Restorative Dental Sciences, Division of Operative Dentistry, College of Dentistry, Jazan University, Jazan 45142, Saudi Arabia; 4 Department of Conservative Dentistry and Endodontics, Saveetha Dental College and Hospitals, Saveetha Institute of Medical and Technical Sciences, Saveetha University, Chennai 600077, India; 5 Division of Community Dentistry, Department of Preventive Dental Sciences, College of Dentistry, Jazan University, Jazan 45142, Saudi Arabia; 6 Dentistry and Oral Health, Department of Rural Clinical Sciences, La Trobe Rural Health School, La Trobe University, Bendigo 3550, Australia; 7 Violet Vines Marshman Centre for Rural Health Research, La Trobe Rural Health School, La Trobe University, Bendigo 3550, Australia; 8 Division of Orthodontics, Department of Preventive Dental Sciences, College of Dentistry, Jazan University, Jazan 45142, Saudi Arabia

**Keywords:** prevalence, child, taurodontism, supernumerary tooth, Saudi Arabia, orthopantomography

## Abstract

Background: This study aimed to ascertain the prevalence of dental anomalies and their ability to estimate sex status. Material and Methods: This cross-sectional radiographic study was based on the evaluation of dental anomalies of Saudi children aged between 5 and 17 years. A total of 1940 orthopantomograms (OPG) were screened, of which 1442 were included. All the OPGs were digitally evaluated with ImageJ software. The demographic variables and dental anomaly findings were subjected to descriptive and comparative statistical analysis. Discriminant function analysis was conducted for sex estimation. *p* value < 0.05 was considered as significant. Results: The mean age of the children in this study was 11.35 ± 0.28 years. At least one dental anomaly was detected in 161 children (11.17%), including 71 males and 90 females. Only 13 children (8.07%) presented with more than one anomaly. The most common dental anomaly detected was root dilaceration (47.83%) followed by hypodontia (31.68%). The least common dental anomaly was infraocclusion (1.86%). The sex prediction accuracy using discriminant function analysis was 62.9% (*p* < 0.01). Conclusion: The prevalence of dental anomalies was 11.17% with root dilaceration and hypodontia being the most common. The role of dental anomalies in sex estimation was found to be ineffective.

## 1. Introduction

Dental anomalies are abnormalities in the color, contour, size, and number of teeth [1]. These anomalies could be related to the number (hypodontia and hyperdontia), size (microdontia and macrodontia), shape (gemination, fusion, concrescence, accessory cusps, dens invaginatus, ectopic enamel, taurodontism, hypercementosis, accessory roots, dilaceration), and structure (amelogenesis imperfecta, dentinogenesis imperfecta, dentin dysplasia, regional odontodysplasia) [2]. Hypodontia, supernumerary teeth, fused teeth, and peg-lateral incisors are the most common dental anomalies observed in children [3]. Many factors account for their etiology, including genetic factors, prenatal and postnatal sequela, and systemic conditions [4]. Disturbances that take place during the morphodifferentiation stage of tooth development lead to the formation of aberrations in tooth number, shape, and position [5]. For the anthropological investigations and therapeutic management of patients, data on dental abnormalities are crucial. Many studies have found a correlation between the prevalence of dental anomalies and dental malocclusion, requiring future orthodontic management [6,7]. Moreover, the anatomical aberrations associated with dental anomalies require special consideration when undertaking restorative, endodontic, and surgical procedures in the oral cavity [1].

Globally, the prevalence of dental anomalies has been reported to be between 3–45% [8,9]. However, the prevalence of these anomalies in primary dentition is significantly low, ranging from 0.4 to 8.1% [10,11,12]. Differences in the prevalence and distribution of dental anomalies according to ethnicity is also apparent from the literature [13]. Studies from Saudi Arabia have found differences in the prevalence rates of dental anomalies between the regions of Saudi Arabia. The differences could be attributed to not only the ethnic variations but also differing methodologies between the studies [14,15,16]. However, no studies were recently conducted among children in the Jazan region.

Malocclusion can result from changes in the pattern of tooth eruption due to dental anomalies, which can also affect how the dental arches are arranged. All of them have the potential to alter dental restorative, endodontic, and surgical procedures [1].

In addition to ethnic differences, teeth features, such as morphology, crown size, and root lengths, could be different between males and females. Jain et al. [17] observed a male preponderance in dental anomalies pertaining to number and size, although not statistically significant. Studies found differences in mesiodistal dimensions of primary [18] and permanent teeth [19] between male and female children and adolescents. Lopez-Lazaro et al. [20] studied the sexual dimorphism of the occlusal features of the first deciduous molar using the geometric morphometric analysis technique and concluded that the maxillary deciduous first molar could aid in sex estimation. Sex estimation using teeth could be a reliable method in young individuals where secondary sexual characteristics of the skeleton are not fully developed [21]. Despite there being studies exploring the differences in tooth dimensions between males and females, to our knowledge, there are no studies that evaluated the possibility of estimating sex in children and adolescents using dental anomalies.

The present study aimed to assess the prevalence of dental anomalies in children from the Jazan region and explores the ability of dental anomalies to estimate sex.

## 2. Materials and Methods

In this cross-sectional study, the authors evaluated the dental anomalies of patients attending the Jazan University dental teaching hospital between January 2017 and March 2019 using their radiographs. Ethical approval was sought for the study from the institutional ethics committee (REC-44/06/468), and informed consent was obtained from all the participants.

### 2.1. Inclusion and Exclusion

Only children of Saudi nationality between 5 and 17 years were included in the study; they all required orthopantomograms (OPG) as part of their routine oral examination and treatment planning. Third molars were not observed due to their tendency for variations in external morphology and position. The exclusion criteria were as follows: positive history of systemic conditions; cleft lip and/or palate; missing tooth/teeth due to caries, trauma, orthodontic management, or incomplete root formation; syndromes likely to cause dental anomalies; and blurred low-quality radiographs.

### 2.2. Sample Size Calculation

A previous study [22] from the Jazan region in Saudi Arabia reported a prevalence of 38% for dental anomalies among adults. A minimum sample of 363 was required to estimate a proportion of 38% with a 95% confidence level and a 5% margin of error. However, a total of 1940 radiographs were available for consideration in this study.

All the OPGs were taken using Orthophos XG Sirona machine (Long Island City, NY, USA, using the following parameters: 65–90 kV, 15 mA, 13 s, 110 mGy cm, effective dose = 21.4 mSv) and digitally evaluated with ImageJ software (National Institutes of Health, Bethesda, MD, USA). The digital images were visualized using optimal lighting, screen brightness, and resolution for anomalies in both primary and permanent dentition.

The OPGs were assessed by two examiners who were general dentists. The general dentists were calibrated by an experienced pediatric dentist. Twenty OPGs were selected randomly to estimate the reliability in identifying dental anomalies on OPGs between the two examiners. The inter-examiner reliability was assessed, and the agreement rate was determined to be excellent (*Kappa* = 0.79). The following six dental anomalies were evaluated: Taurodontism was assessed based on the criteria by Schiffman and Chanannal [23]. Root dilaceration was assessed by measuring the root angle deviating from the normal axis of the tooth [24]. Dental anomalies in shape and number (peg shape, hypdontia, supernumerary) were evaluated visually according to Kreiborg et al. [25]. Infraocclusion was also recorded, as it indicates that the tooth might be ankylosed, and was measured by adopting the methodology introduced by Odeh et al. [26].

### 2.3. Statistical Analysis

The demographic characteristics (sex and age) from the patient records and evaluated image findings were recorded in a data extraction sheet. The data were subjected to descriptive and analytical statistics using the IBM SPSS Statistics for Windows software program, version 23.0 (IBM Corp., Armonk, NY, USA). Chi-square tests and Fisher’s exact tests were used to compare the prevalence of dental anomalies according to age, sex, dental arch and side. Logistic regression analysis was performed to predict the sex of the individuals with dental anomalies, with arch and side as the independent variables. Discriminant function analysis (DFA) was performed to derive a discriminant regression equation for sex estimation. We produced a discriminant function based on the presence or absence of each dental anomaly (rated as “1” in the presence of a dental anomaly, otherwise rated as “2”). Six dental anomalies were included in the DFA. A *p*-value < 0.05 was considered significant.

## 3. Results

A total of 1940 OPGs were screened, of which 1442 children (690 males and 792 females) fulfilled the inclusion criteria. At least one dental anomaly was detected in 161 children (11.17%), including 71 males and 90 females. The mean age of the children in this study was 11.35 ± 0.28 years. Only 13 children (8.08%) presented with more than one anomaly. None of the children had dental anomalies in the primary dentition. Table 1 shows the demographic profile of the final sample and the distribution of dental anomalies. The most common dental anomaly detected was root dilaceration (47.83%), followed by hypodontia (31.68%). The least common dental anomaly seen was infraocclusion (1.86%). The most common congenitally missing tooth (hypodontia) was the left mandibular premolars (27.3%).

Table 2 shows the distribution of anomalies in the final sample according to sex, age groups, side, and jaw. Root dilaceration in males (59.15%) was predominantly more prevalent compared to females (38.2%), *p* < 0.05. On the other hand, the prevalence of hypodontia was significantly higher in females (41.57%) than males (20.00%). Root dilacerations were also significantly more prevalent in children >10 years of age (64.04%) and mandible (63.25%) when compared to those ≤10 years of age (27.4%) and maxilla (0%). Hypodontia was significantly more prevalent in children ≤10 years of age (48.61%) and mandibular region (24.79%) in comparison to ≤10 age group and maxillary region, respectively. Supernumerary teeth were significantly more prevalent in the maxillary (35.29%) than the mandibular region, of which mesiodens (not presented in tables) comprised the majority. Dental anomalies were more prevalent in the mandible region than the maxillary region. Particularly, peg-shape teeth were significantly more prevalent in the mandibular region (7 patients, 6.09%) compared to the maxillary region (1 patient, 2.94%). Table 3 presents the frequency of different anomaly combinations in children, of which the combination of taurodontism with root dilacerations was the most predominant.

Table 4 depicts the prevalence of dental anomalies among the age groups according to sex. Hypodontia was significantly more prevalent in females than males among the ≤10 years age group. Table 5 displays the results of logistic regression analysis. Those with root dilaceration were 2.19 times (95% CI: 1.16–4.14) more likely to be females than males.

The unstandardized coefficients were used to generate the discriminant function equation. The discriminant model for sex estimation was obtained as follows: sex = −12.743 − 0.182 (taurodontism) + 1.372 (root dilaceration) − 0.412 (peg shaped) − 0.534 (hypodontia) + 1.675 (supernumerary teeth) + 4.794 (infraocclusion).

This discriminant model could be helpful for sex estimation in the new data using the adjusted canonical centroids of −0.323 to 0.261. If the value obtained is close to −0.323, the proposed sex is likely a male, but if it is relatively close to 0.261, it will probably be a female.

The output of the equality test of mean difference for male and female parameters shows that two out of six predictors, i.e., root dilaceration and hypodontia, were statistically significant (Table 6). When tested with the present data, this model derived an ‘F’ likelihood ratio test with a model accuracy of 62.9% (Table 6 and Table 7).

The prediction using this model is statistically significant (*p* < 0.01); however, a 62.9% predictability into group membership seems relatively low, with a better prediction for males (73.2%) than females (54.5%). Based on these results, it can be concluded that cautious predictions about group membership should be made using this model.

## 4. Discussion

Saudi Arabia has a diverse population with various ethnicities. Many studies have been conducted in various regions of the Kingdom to ascertain the prevalence rate of dental anomalies, but as expected, they yielded differing results with prevalence ranging from 3.2% to 56% [27,28]. The disparities can be explained by the variations in the sample size, sampling methods, and ethnicity. Any variation in the shape of the teeth, function, and position plays a significant role in the study of human evolution [29]. This study was carried out among children in the Jazan region utilizing digital technology to assess the prevalence of dental anomalies in children. In a recent study conducted on the Iranian population recruited from three private clinics, the prevalence rate of dental anomalies was found to be 28.06% [8]. The present study was conducted in a teaching dental hospital catering to the needs of people from the surrounding suburban region, but the sample might not be representative of the population of the whole Jazan region. However, the data from this study could serve as a baseline for future studies from the region.

Our study’s prevalence of dental anomalies was 11.2%, similar to the findings from a study conducted in Yemen [30] but varied from a previous study among the adult population of the Jazan region [22]. The similarity of the findings can be explained by the proximity of Jazan to the neighboring country, sharing a similar ethnic background between the regions. The difference between our findings with the previous study [22] can be explained by the fact that their study was exclusively conducted on an adult population. Moreover, their study included anomalies such as rotation and impaction, which could have resulted in an increased estimation of the prevalence. On the other hand, overall anomaly prevalence observed in our study was higher than the study by Salem [27] involving children of the Jazan region, which was conducted in 1989. The difference in prevalence could probably be because he assessed relatively fewer anomalies. More females presented with dental anomalies than males, but it was not statistically significant. This result complies with results from previous studies [7,31,32].

Root dilaceration was present in 5.3% of the total sample, which is congruent with the data from the Yemeni population by Aldhorae et al. [33], relatively close to the findings of Vani et al. [22] and Bawazir et al. [32] but disagrees with that of Al-Halal et al. [28] and AlHumaid et al. [14], where they reported a high prevalence of around 30%. The contradiction between the findings could be attributed to the inclusion of third molars in their study; third molars tend to present high prevalence of root dilacerations up to 30.92%, especially in the maxillary arch [34].

In our study, hypodontia was detected in 3.5%, which falls within the limits (0.15–16.2%) estimated in the literature review by Rakhshan [35]. It is almost similar to the results from Shafi et al. [36] in an Indian population [37] but in contrast to other studies from various regions of Saudi Arabia where they reported a higher prevalence [7,14,31], ranging from 20–25.7%. The differences can be explained by the variation in the ethnicity of the study population and inclusion/exclusion of third molars. We found that more females presented with dental anomalies than males, and mandibular second premolars were the most affected teeth. This is congruent with data documented by other studies from Saudi Arabia [14,15,36]. Taurodontism was observed in 1.3% of subjects, which is consistent with findings from other Saudi studies [15,28].

Many studies from Saudi Arabia reported the prevalence rate of supernumerary teeth in the range of 1–1.8% [14,22,32]. These findings are similar to a prevalence of 1.2% observed in the current study. Although not observed in our patients, mesiodens were the most common supernumerary tooth detected, which is predominant in the maxilla [14,15]. Previous studies from Saudi Arabia [22,27] recorded the prevalence of peg-shaped teeth to be less than 1%, which is congruent to that of our study (0.5%).

Infraocclusion was detected in only three of our patients (0.2%), which is meagre when compared to the results of studies conducted in other regions of Saudi Arabia [38,39]. Only a few studies have evaluated this anomaly. Moreover, in our study, we have evaluated this anomaly using a validated assessment criteria yielding less values.

A higher prevalence of dental anomalies was observed in the mandibular region, and the peg-shape anomaly was more common among those aged >10 years. This can be explained by the period at which this anomaly is appreciated by the parents of children, resulting in them bringing their child into the clinic for dental treatment. However, a large sample size and evaluation of radiographs by two calibrated examiners has rendered the data highly reliable.

The main limitation of the study was that patients seeking treatment were the study sample, and hence the results cannot be generalized to the general population. Further investigations with a large sample size representing the entire Jazan community would yield better prevalence data. However, the location of the teaching hospital facilitated a larger catchment area, and it was easily accessible for people commuting from various villages.

To the best of our knowledge, this is the first study to attempt sex estimation based on dental anomalies. We produced a discriminant function based on the presence or absence of each dental anomaly. The expression of sexual dimorphism from dental anomalies resulted in 62.9% predictability. These findings indicate that the produced sex discriminant function is ineffective for sex estimation and should be used cautiously. Further work using data from large and diverse population samples is warranted to further evaluate the ability of dental anomalies to estimate sex.

## 5. Conclusions

The prevalence of dental anomalies among the sample of children visiting a teaching hospital in the Jazan region in Saudi Arabia was 11.17%. Root dilaceration and hypodontia were the most common, while infraocclusion was the least prevalent anomaly. Using dental anomalies to estimate sex was found to be ineffective.

## Figures and Tables

**Table 1:** Descriptive statistics of demographic data and dental anomalies (*n* = 161).

Variable	Category	*n* (%)	Mean ± SD
Sex	Males	71 (44.10)	
	Females	90 (55.90)	
Dental anomaly	Taurodontism	18 (11.18)	
	Root Dilaceration	77 (47.83)	
	Peg Shape	7 (4.35)	
	Hypodontia	51 (31.68)	
	Supernumerary Tooth	18 (11.18)	
	Infraocclusion	3 (1.86)	
	Two or more anomalies	13 (8.08)	
Side of anomaly	Right	39 (24.68)	
	Left	27 (17.09)	
	Both	92 (58.23)	
Location of anomaly	Maxilla	34 (21.38)	
	Mandible	115 (72.33)	
	Both	10 (6.29)	
Age			11.35 ± 0.28

**Table 2:** Distribution of dental anomalies among sex and age (*n* = 161).

Dental Anomaly	Sex (*n* = 159)		Age (*n* = 161)		Side (*n* = 158)			Jaw (*n* = 159)		
	Male	Female	≤10	>10	Right	Left	Both	Max	Man	Both
Taurodontism	7 (10.0)	11 (12.36)	7 (9.59)	11 (12.50)	1 (2.56)	4 (14.81)	13 (14.13)	1 (2.94)	15 (13.04)	2 (20.0)
Root Dilaceration	42 (60.0)	34 (38.20) *	20 (27.40)	57 (64.77) *	16 (41.03)	10 (37.04)	51 (55.43)	0	74 (64.35)	3 (30.0) **
Peg Shape	2 (2.86)	5 (5.62)	1 (1.37)	6 (6.82)	2 (5.13)	1 (3.07)	4 (4.35)	6 (17.65)	0	1 (10.0) **
Hypodontia	14 (20.0)	37 (41.57) *	35 (47.95)	16 (18.18) *	12 (30.77)	10 (37.04)	28 (30.43)	16 (47.06)	28 (24.35)	6 (60.0) **
Supernumerary Tooth	10 (14.29)	7 (7.87)	10 (13.70)	8 (9.09)	8 (20.51)	3 (11.11)	6 (6.52)	12 (35.29)	4 (3.48)	2 (20.0) **
Infraocclusion	1 (1.43)	2 (2.25)	2 (2.74)	1 (1.14)	0	0	3 (3.26)	0	2 (1.74)	1 (10.0)
More than one anomaly	5 (7.14)	8 (8.99)	3 (4.11)	10 (11.36)	0	0	13 (14.13)	1 (2.94)	7 (6.09)	5 (0.50) **

* Chi-square test is significant at *p* < 0.05. ** Fisher’s exact test is significant at *p* < 0.05.

**Table 3:** Frequency of different anomaly combinations.

Anomalies	Frequency
Taurodontism + Root Dilaceration	6
Root Dilaceration + Supernumerary Tooth	2
Taurodontism + Peg Shape	1
Root Dilaceration + Hypdontia	1
Peg Shape + Hypodontia	1
Hypodontia + Supernumerary Tooth	2

**Table 4:** Prevalence of different anomalies among sex and age groups.

	Whole Sample *n* = 1442			≤10-Year-Old Children			>10-Year-Old Children	
	*n* (%)	Males *n* = 690	Females *n* = 752	*n* (%)	Males *n* = 27	Females *n* = 45	Males *n* = 43	Females *n* = 44
Taurodontism	18 (1.25)	7 (1.02)	11 (1.46)	18 (1.25)	2 (7.41)	5 (11.11)	5 (11.63)	6 (13.64)
Root Dilaceration	76 (5.27)	42 (6.09)	34 (5.59)	77 (5.34)	11 (40.74)	9 (20.0)	31 (72.1)	25 (56.8)
Peg Shape	7 (0.49)	2 (0.29)	5 (0.67)	7 (0.49)	1 (3.70)	0	1 (2.33)	5 (11.37)
Hypodontia	51 (3.54)	14 (2.03)	37 (4.92)	51 (3.54)	8 (3.00)	27 (60.0) *	6 (13.95)	10 (22.7)
Supernumerary Tooth	17 (1.18)	10 (1.45)	7 (0.93)	18 (1.24)	6 (2.22)	3 (6.67)	4 (9.30)	4 (9.09)
Infraocclusion	3 (0.21)	1 (0.15)	2 (0.27)	3 (0.21)	1 (3.70)	1 (2.22)	0	1 (2.27)
More than anomaly	13 (0.90)	5 (0.73)	8 (1.06)	13 (0.90)	2 (7.41)	1 (2.22)	3 (6.98)	7 (15.91)

* Fisher exact test is significant at *p* < 0.05.

**Table 5:** Ordinal logistic regression with sex as the outcome variable and dental anomalies, arch and side being the explanatory variables (*n* = 161).

		OR	CI 95%
Dental anomalies	Taurodontism	0.77	(0.28, 2.09)
	Root Dilaceration	2.19	(1.16, 4.15) *
	Peg Shape	0.48	(0.09, 2.56)
	Hypodontia	0.37	(0.18, 0.77)
	Supernumerary Tooth	2.12	(0.78, 5.79)
	Infraocclusion	Reference	
Arch	Maxilla	0.75	(0.37, 1.53)
	Mandible	1.17	(0.55, 2.48)
	Both	Reference	
Side	Right	0.85	(0.42, 1.72)
	Left	0.91	(0.41, 2.01)
	Both	Reference	

* Logistic regression is significant at *p* < 0.05.

**Table 6:** Tests of equalities of group means.

	Wilks’ Lambda	F	df1	df2	Sig.
Taurodontism	0.99	0.27	1	157	0.60
Root dilaceration	0.96	6.06	1	157	0.02 *
Peg shaped	0.99	0.76	1	157	0.39
Hypodontia	0.95	7.68	1	157	0.01 *
Supernumerary teeth	0.99	2.23	1	157	0.14
Infraocclusion	0.99	1.24	1	157	0.27

* Statistically significant (*p* < 0.05).

**Table 7:** Percentage predictability for group membership.

		Sex	Predicted Group Membership		Total
			Male	Female	
Original ^a^	Count	Male	53	18	71
		Female	40	48	88
	%	Male	74.6	25.4	100.0
		Female	45.5	54.5	100.0
Cross-validated ^b^	Count	Male	52	19	71
		Female	40	48	88
	%	Male	73.2	26.8	100.0
		Female	45.5	54.5	100.0

^a^ 63.5% of original grouped cases correctly classified; ^b^ 62.9% of cross-validated grouped cases correctly classified.

## Data Availability

Not applicable.
